# Comparison of outcomes between preservation or division of the uterine round ligament in laparoscopic groin hernia repair in females: a meta-analysis and trial sequential analysis

**DOI:** 10.1007/s10029-023-02917-6

**Published:** 2024-01-02

**Authors:** W. Zhong, L. Zhang, J. Q. Zhong, X. P. He, H. Q. Liu, W. Q. Zhu, C. F. Fang

**Affiliations:** Department of Gastrointestinal Surgery, The Ganzhou People’s Hospital, Ganzhou, China

**Keywords:** Groin hernia, Uterine round ligament, Laparoscopic repair, Females

## Abstract

**Purpose:**

The purpose of this study was to perform a meta-analysis comparing the short-term and long-term outcomes in laparoscopic groin hernia repair with or without preservation of the uterine round ligament (URL) in females.

**Methods:**

We searched several databases including PubMed, Web of Science, Cochrane Library, and and CNKI databases. This meta-analysis included randomized clinical trials, and retrospective comparative studies regarding preservation or division of the URL in laparoscopic groin hernia repair in females. Outcomes of interest were age, BMI, type of hernia, type of surgery, operating time, estimated blood loss, time of hospitalization, seroma, concomitant injury, mesh infection, recurrence, uterine prolapse, foreign body sensation, chronic pain, and pregnancy. Meta-analyses and trial sequential analysis were performed with Review Manager v5.3 and TSA software, respectively.

**Results:**

Of 192 potentially eligible articles, 9 studies with 1104 participants met the eligibility criteria and were included in the meta-analysis. There were no significant difference in age (MD-6.58, 95% CI − 13.41 to 0.24; P = 0.06), BMI (MD 0.05, 95%CI − 0.31 to 0.40; P = 0.81), blood loss (MD-0.04, 95% CI − 0.75 to 0.66; P = 0.90), time of hospitalization (MD-0.22, 95% CI-1.13 to 0.69; P = 0.64), seroma (OR 0.71, 95% CI 0.41 to 1.24; P = 0.23), concomitant injury (OR 0.32, 95% CI 0.01 to 8.24; P = 0.68), mesh infection (OR 0.13, 95% CI 0.01 to 2.61; P = 0.18), recurrence (OR 1.13, 95% CI 0.18 to 7.25; P = 0.90), uterine prolapse(OR 0.71, 95% CI 0.07 to 6.94; P = 0.77), foreign body sensation (OR 1.95, 95% CI 0.53 to 7.23; P = 0.32) and chronic pain(OR 1.03 95% CI 0.4 to 2.69; P = 0.95). However, this meta-analysis demonstrated a statistically significant difference in operating time (MD 6.62, 95% CI 2.20 to 11.04; P = 0.0003) between the preservation group and division group. Trial sequential analysis showed that the cumulative Z value of the operating time crossed the traditional boundary value and the TSA boundary value in the third study, and the cumulative sample size had reached the required information size (RIS), indicating that the current conclusion was stable.

**Conclusion:**

In summary, laparoscopic groin hernia repair in women with the preservation of the round uterine ligament requires a longer operating time, but there was no advantage in short-term or long-term complications, and there was no clear evidence on whether it causes infertility and uterine prolapse.

## Introduction

Groin hernia is a prevalent condition in the field of general surgery, with a higher incidence rate among males. However, the occurrence of female groin hernia remains relatively low, with incidence of 0.3% [1]. With the popularity of laparoscopic surgery, more and more women are undergoing laparoscopic groin hernia repair [2, 3]. The international guidelines for inguinal hernia management proposed by the HerniaSurge Group in 2018 recommend laparoendoscopic repair with mesh implantation for women with primary inguinal hernias [4]. Due to the tight adhesion of the URL to the peritoneum, it is difficult to completely separate the URL in laparoscopic surgery, so the treatment of URL was controversial. Renshaw’s study which included 1365 women with groin hernias who underwent traditional open surgery, laparoscopic surgery, and robotic surgery showed that there were no statistical difference in postoperative complications and recurrence rates between preservation and division groups, and they also found that the division group experienced less pain at 6 months than the preservation group [5]. However, with the gradual proficiency of laparoscopic techniques and the deepening of the understanding of the function of URL, many experts began to prefer to retain URL [6]. At present, there is a lack of multicenter, large sample size randomized controlled studies on the preservation of URL in female groin hernia surgery, and the preservation of URL often depends on the personal preference of the surgeon. We reviewed a lot of literature, and there is currently no meta-analysis on whether URL is preserved during laparoscopic groin hernia repair in females. Therefore, this paper will conduct a meta-analysis on the treatment of URL in laparoscopic repair of groin hernia in females, which will provide references for the treatment of URL by surgeons in the future.

## Methods

### Literature search

The databases of PubMed, Web of Science, Cochrane Library, Embase, and CNKI were searched respectively. Literature indexed from January 1, 1980 to June 1, 2023. The following search terms were used: “Female hernias”, “Round ligament”, “Round ligament of uterus”, “Laparoscopic”.

### Inclusion and exclusion criteria

Inclusion criteria: (1) Studies in any language can be included; (2) Female patients undergoing laparoscopic groin hernia tension-free repair; (3) Randomized controlled or non-randomized controlled studies that reported age, BMI, operating time, estimated blood loss, time of hospitalization, seroma, concomitant injury, mesh infection, recurrence, uterine prolapse, foreign body sensation, chronic pain, pregnancy and other indicators. Exclusion criteria: (1) Abstracts, letters, expert opinions, systematic reviews and case reports are excluded; (2) Excluded studies that included traditional open surgery; (3) The studies of high ligation of hernia sac alone was excluded. The researchers first screened by title and abstract, and then screened by reading the full text. If studies are replicated, only the most recent studies were included.

### Data extraction

Two researchers (ZW, FCF) independently extracted the following data from the included studies: first author, date of publication, study period, number of patients, country, study design, age, BMI, type of hernia, type of surgery, operating time, estimated blood loss, time of hospitalization, seroma, concomitant injury, mesh infection, recurrence, uterine prolapse, foreign body sensation, chronic pain, and pregnancy. If there was a disagreement during data collection, it would be resolved by a third researcher (ZWQ).

### Assessment of methodological quality of included studies

On the basis of the standards described in the Cochrane Collaboration Handbook, risk of bias of studies contained in this review will be evaluated by all the authors [7]. Disagreements between authors were resolved through discussion.

### Statistical analysis

Meta-analysis was performed using Review Manager 5.2 (The Cochrane Collaboration, Oxford, UK). Heterogeneity was assessed using Cochran’s Q test and *I*^*2*^ statistics, and *P* < 0.1 was considered to indicate statistical significance. Sensitivity analysis was also performed by removing one study at a time and repeating the meta-analysis. Funnel plots were used to assess potential publication bias [8].

TSA was utilized to determine the required sample size for the meta-analysis and address the limitations of traditional meta-analysis. Some “positive” meta-analysis results may be attributable to random error; when the number of trials in a metaanalysis and the sample size of patients are small, random error may yield erroneous results [9–11]. In this study, the researchers used the version 0.9.5.10 Beta TSA software to perform the analysis. The risk of type I and II errors was set to 5% and 20%, respectively. Concurrently, the alpha spending function, continuously monitoring boundaries, and evaluation of invalid boundary areas were estimated [12].

## Results

### Search results

**Fig. 1:**
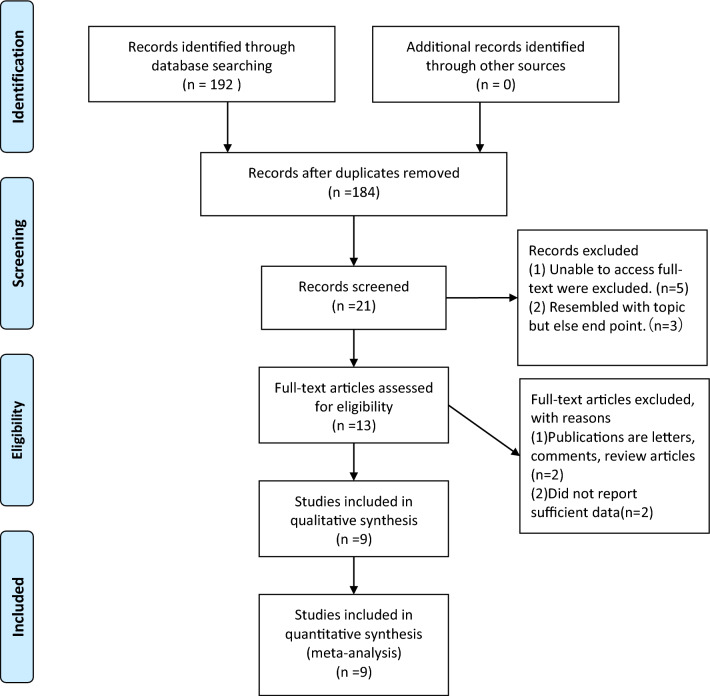
PRISMA systematic review fow diagram

Our original search strategy yielded 192 potential studies (Fig. 1). After reading the title and abstract, 171 studies were excluded, leaving 21 studies, and after reading the full text again, 12 studies were excluded, of which 5 were excluded because the full text was not available, 3 studies did not report the required results, 2 were letters or comments, and 2 did not report sufficient data. In the end, we included 9 studies published between 2015 and 2023 [13–21], including 6 non-randomized controlled studies [13, 15, 17–19, 21] and 3 randomized controlled studies [14, 16, 20]. The 9 studies included 1104 patients, 578 of whom had the URL preserved and 526 of whom had the URL transected.

### Study characteristics and quality

**Fig. 2:**
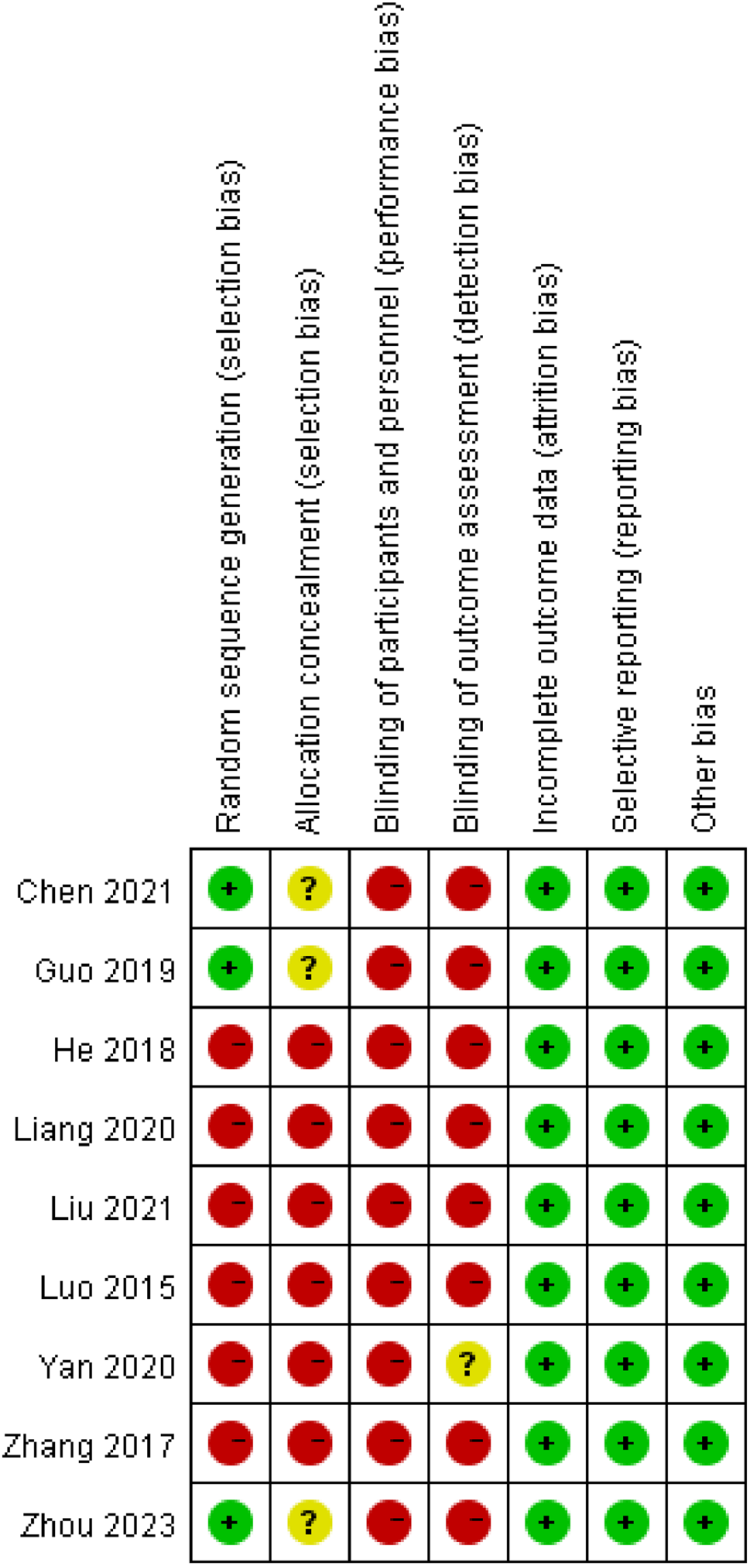
Risk of bias summary

The 9 included studies were all from China, including 8 from mainland China and 1 from Hong Kong, 5 published in English and 4 published in Chinese, with sample sizes ranging from 34 to 393 (Table 1). The risk of bias summary is presented in Fig. 2.

**Table 1:** Characteristics of trials included in the meta-analysis

Study	Publication year	Country	Study design	N	Treatment (n)	
					Preservation group	Division group
Luo [13]	2015	China	Retro	34	25	9
Zhang [14]	2017	China	RCT	37	19	18
He [15]	2018	China	Retro	316	152	164
Guo [16]	2019	China	RCT	62	36	26
Luk [17]	2020	China	Retro	77	25	52
Liang [18]	2020	China	Retro	41	21	20
Liu [19]	2021	China	Retro	393	218	175
Chen [20]	2021	China	RCT	60	30	30
Zhou [21]	2023	China	Retro	84	52	32

### Age

**Fig. 3:**
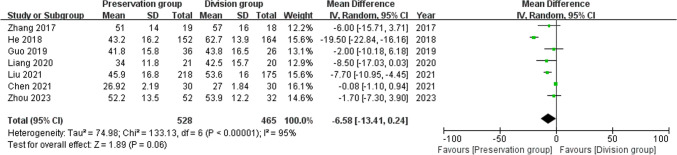
Forest plot of age between the preservation group and the division group

Data on age from 7 studies, including 984 patients (528 preservation group and 465 division group) [14–16, 18–21], with high heterogeneity, and were analyzed using a random-effects model (I^2^ = 95%, P < 0.00001). There was no significant difference in age between the preservation group and the division group (MD-6.58, 95% CI − 13.41 to 0.24; P = 0.06; Fig. 3).

#### BMI

**Fig. 4:**
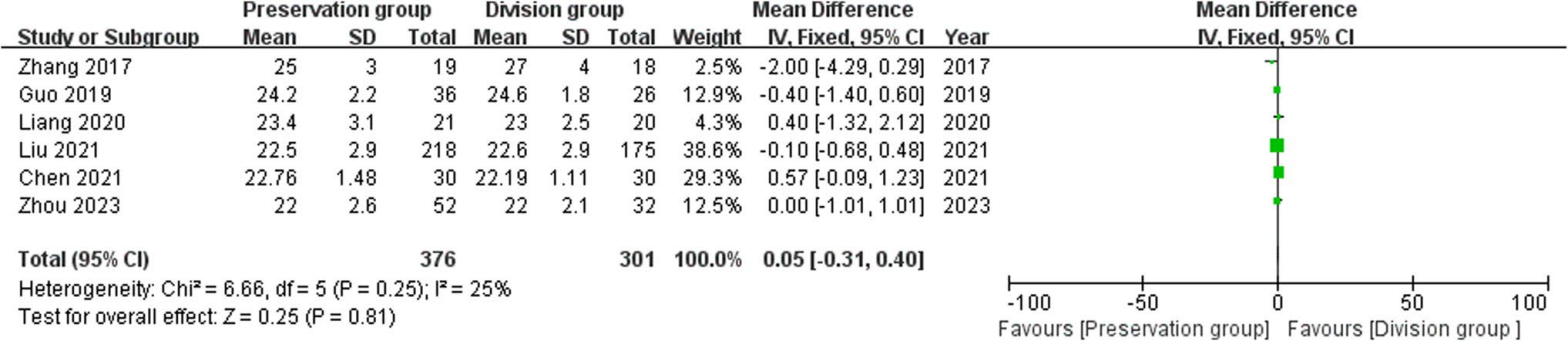
Forest plot of BMI between the preservation group and the division group

6 studies provided data regarding BMI (376 preservation group and 301 division group) [14, 16, 18–21]. There was low heterogeneity among these studies (I^2^ = 25%, P = 0.25) and a fixed-effect model was used for meta-analysis. In the pooled data, there was no significant difference in the BMI between the groups (MD 0.05, 95%CI − 0.31 to 0.40; P = 0.81; Fig. 4).

### Operating time

**Fig. 5:**
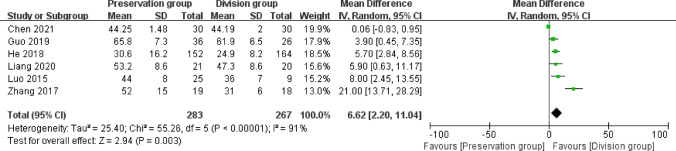
Forest plot of operating time between the preservation group and the division group

Time of operation was reported in 6 studies involving 490 patients (253 preservation group and 237 division group) [13–16, 18, 20], with high heterogeneity (I^2^ = 91%, P < 0.001), analyzed using a random-effects model. There was a statistically significant difference in operating time of operation between the groups (MD 6.62, 95% CI 2.20 to 11.04; P = 0.0003; Fig. 5).

### Blood loss

**Fig. 6:**
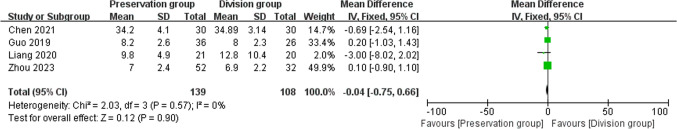
Forest plot of blood loss between the preservation group and the division group

Blood loss was reported in 4 studies involving 247 patients (139 preservation group and 108 division group) [16, 18, 20, 21] with low heterogeneity and analyzed using a fixed-effect model (I^2^ = 0%, P = 0.57). There was no significant difference in blood loss between the preservation group and division group (MD-0.04, 95% CI − 0.75 to 0.66; P = 0.90; Fig. 6).

### Time of hospitalization

**Fig. 7:**
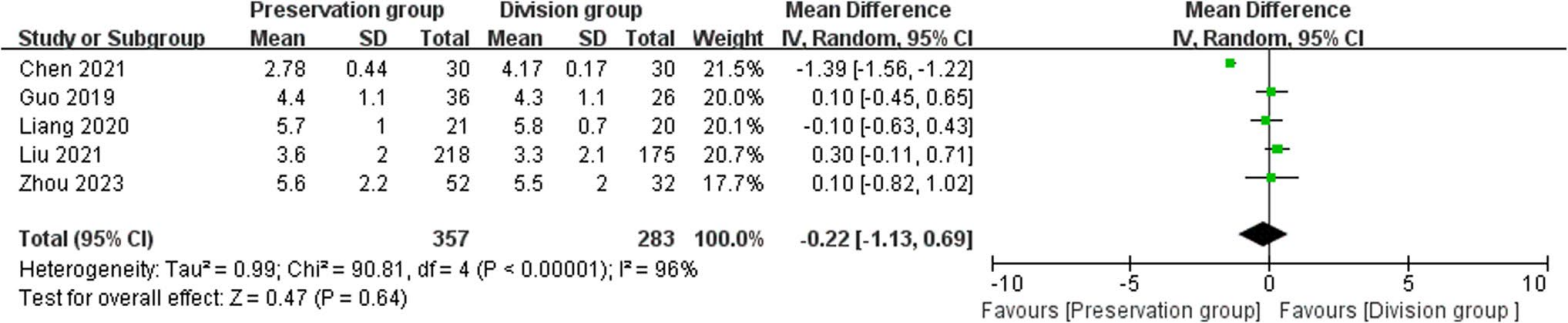
Forest plot comparing hospitalization duration between the preservation and division groups

5 studies reported time of hospitalization, including 640 patients (357 preservation group and 283 division group) [16, 18–21], with high heterogeneity, and were analyzed using a random-effects model (I^2^ = 96%, P < 0.00001). There was no significant difference in time of hospitalization between the preservation group and division group (MD-0.22, 95% CI-1.13 to 0.69; P = 0.64; Fig. 7).

### Seroma

**Fig. 8:**
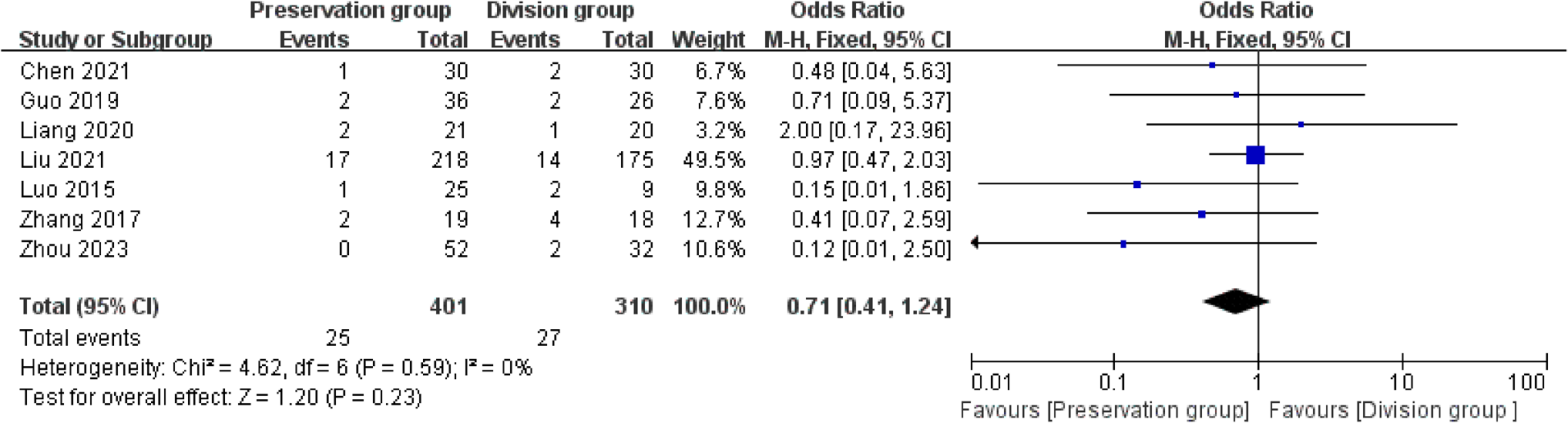
Forest plot compares the occurrence of seroma between the preservation group and the division group

7 studies provided data regarding seroma (401 preservation group and 310 division group) [13, 14, 16, 18–21]. There was low heterogeneity among these studies (I^2^ = 0%, P = 0.59) and a fixed-effect model was used for meta-analysis. In the pooled data, there was no significant difference in the seroma formation between the groups (OR 0.71, 95% CI 0.41 to 1.24; P = 0.23; Fig. 8).

### Concomitant injury

**Fig. 9:**
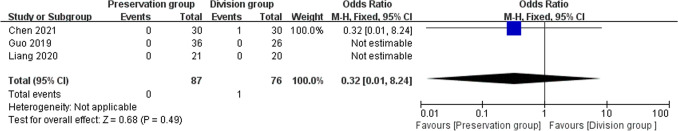
Forest plot compares the occurrence of concomitant injury between the preservation group and the division group

3 studies reported concomitant injury as a complication indicator, including 163 patients (87 preservation group and 76 division group) [16, 18, 20], which could not identify heterogeneity and were analyzed using a random effects model. There was no significant difference in the concomitant injury rate between the groups (OR 0.32, 95% CI 0.01 to 8.24; P = 0.68; Fig. 9).

### Mesh infection

**Fig. 10:**
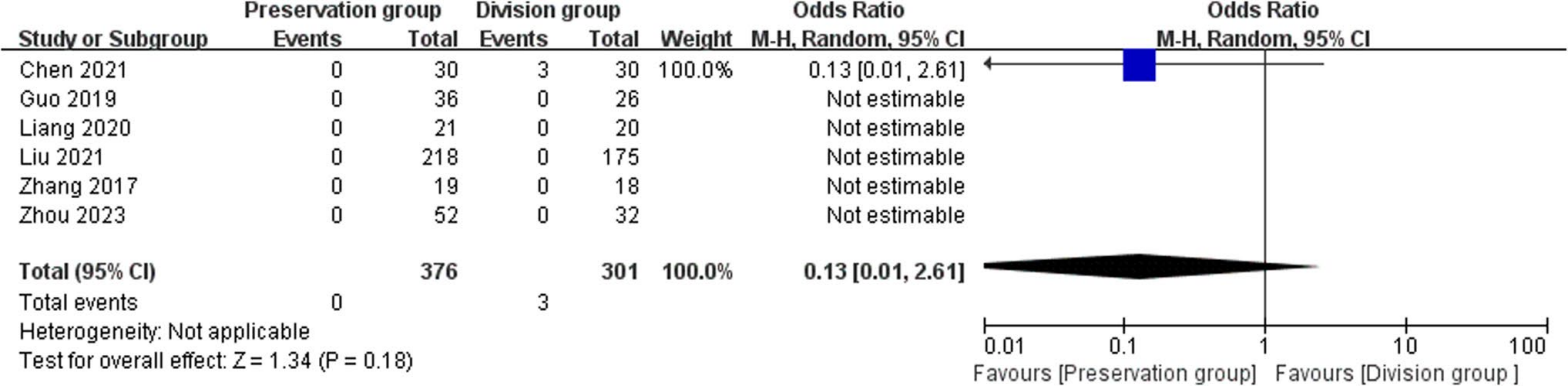
Forest plot compares the occurrence of mesh infection between the preservation group and the division group

6 studies reported mesh infection, including 677 patients (376 preservation group and 301 division group) [14, 16, 18–21], which could not identify heterogeneity and were analyzed using a random-effects model. There was no significant difference in the mesh infection rate between the groups (OR 0.13, 95% CI 0.01 to 2.61; P = 0.18; Fig. 10).

### Recurrence

**Fig. 11:**
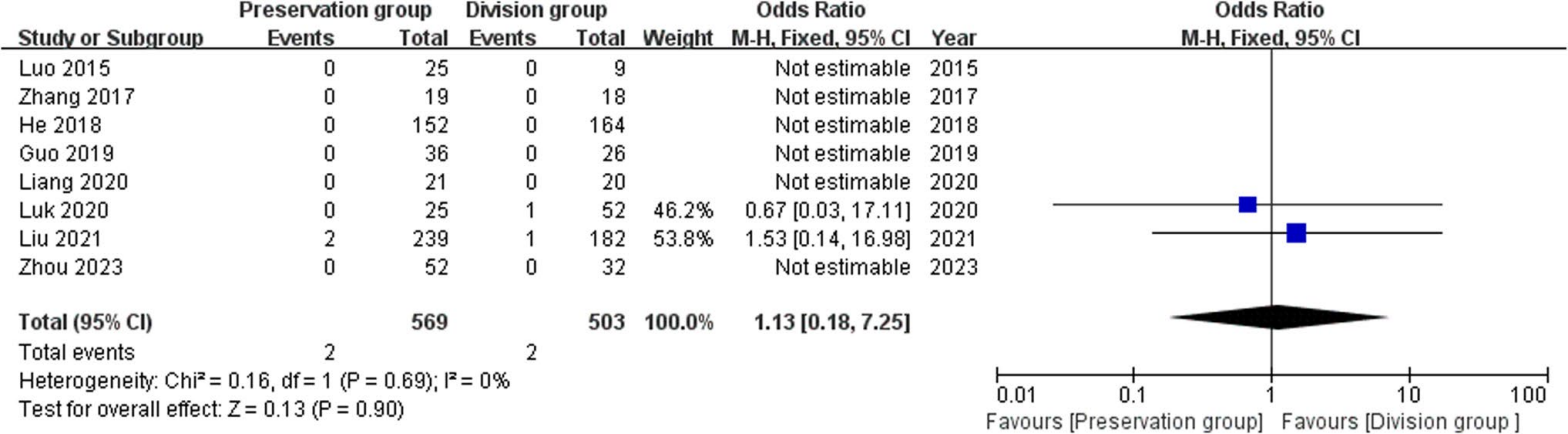
Forest plot comparing recurrence rates between the preservation group and the division group

8 studies reported postoperative recurrence, including 1072 patients (569 preservation group and 503 division group) [13–19, 21], without heterogeneity, and were analyzed using a fixed-effect model (I^2^ = 0%, P = 0.69). There was no significant difference in recurrence rate between the groups (OR 1.13, 95% CI 0.18 to 7.25; P = 0.90; Fig. 11).

### Uterine prolapse

**Fig. 12:**
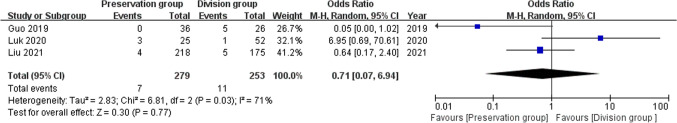
Forest plot comparing uterine prolapse rates between the preservation group and the division group

3 studies provided data regarding uterine prolapse (279 preservation group and 253 division group) [16, 17, 19]. There was high heterogeneity among these studies (I^2^ = 71%, P = 0.03) and a random-effects model was used for meta-analysis. In the pooled data, there was no significant difference in the rate of uterine prolapse between the groups (OR 0.71, 95% CI 0.07 to 6.94; P = 0.77; Fig. 12).

### Foreign body sensation

**Fig. 13:**
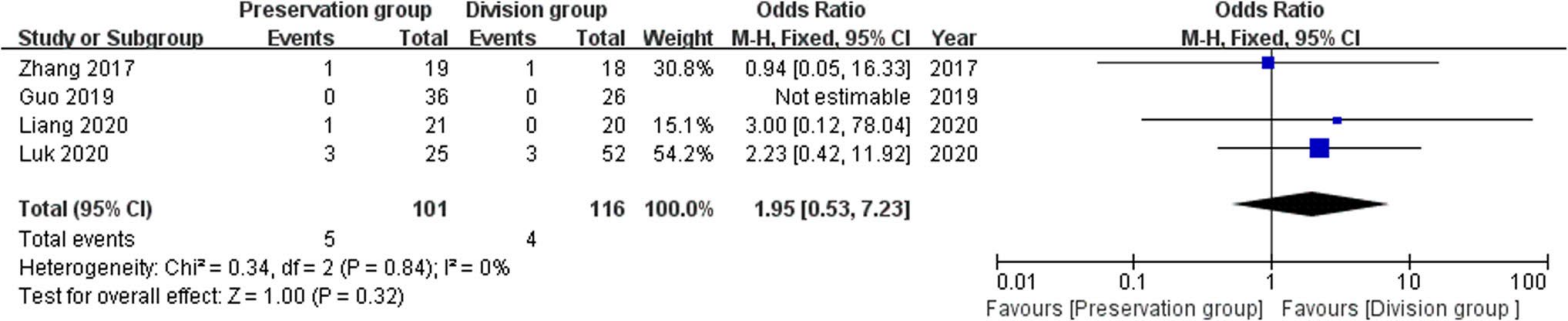
Forest plot compares the occurrence of foreign body sensation between the preservation group and the division group

4 studies reported foreign body sensation as an indicator of complications, including 217 patients (101 preservation group and 116 division group) [14, 16–18], without heterogeneity, and were analyzed using a fixed-effect model (I^2^ = 0%, P = 0.84). There was no significant difference in the rate of foreign body sensation between the groups (OR 1.95, 95% CI 0.53 to 7.23; P = 0.32; Fig. 13).

### Chronic pain

**Fig. 14:**
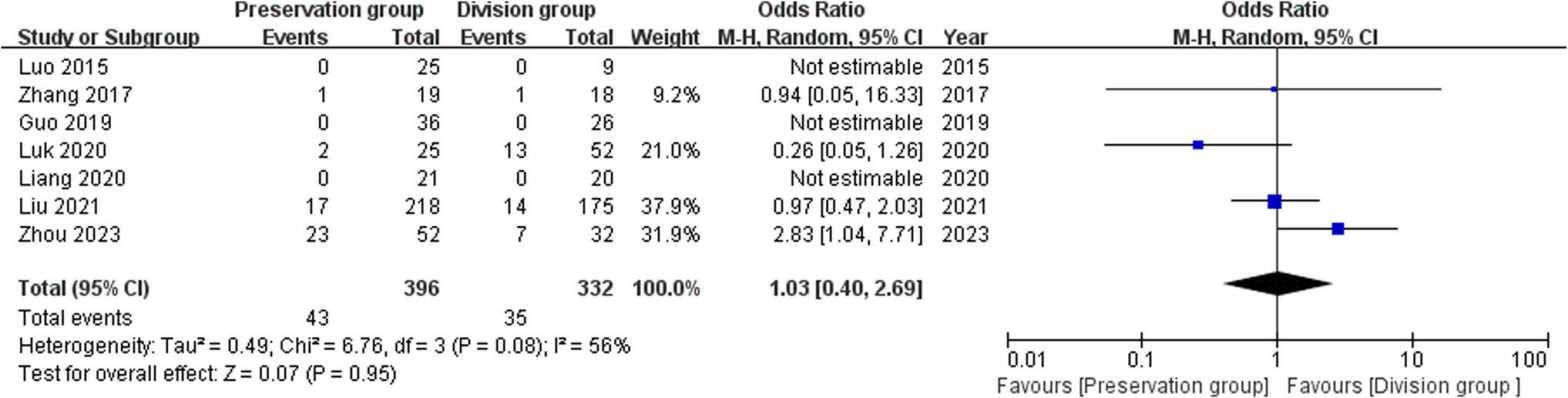
Forest plot compares the occurrence of chronic pain between the preservation group and the division group

7 studies reported chronic pain as an indicator of complications, including 728 patients (396 preservation group and 332 division group) [13, 14, 16–19, 21]. There was medium heterogeneity among these studies (I^2^ = 56%, P = 0.08) and a random-effects model was used for meta-analysis. There was no significant difference in the rate of chronic pain between the groups (OR 1.03 95% CI 0.4 to 2.69; P = 0.95; Fig. 14).

### Trial sequential analysis

**Fig. 15:**
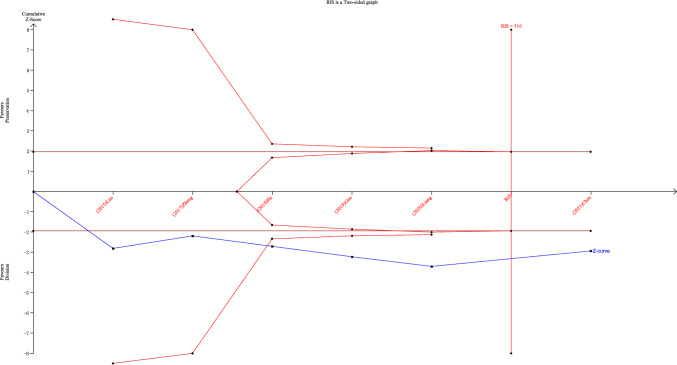
Trial sequential analysis (TSA) curve for operating time

Trial sequential analysis showed that the cumulative Z value of the operating time crossed the traditional boundary value and the TSA boundary value in the third study, and the cumulative sample size had reached the required information size (RIS), indicating that the current conclusion was stable ( Fig. 15).

### Sensitivity analysis and publication bias

**Fig. 16:**
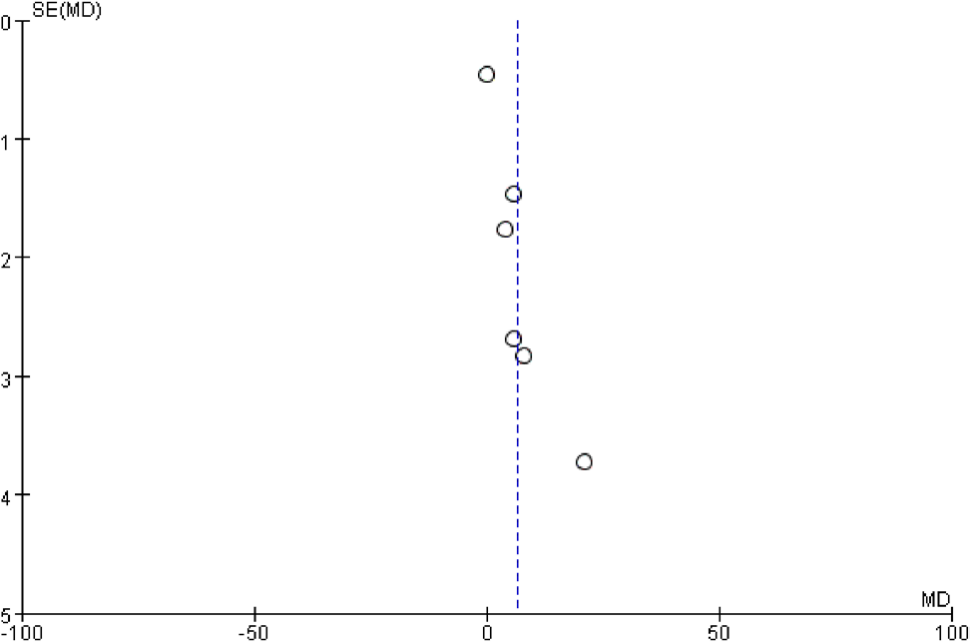
Funnel plot of studies reporting on operating time

For each of the meta-analyses described above, similar results were obtained after removing each study individually (Table 2). For each of the meta-analyses, risk of publication bias was assessed using funnel plots, which did not indicate severe publication bias; as an example, the funnel plot for the meta-analysis of operating time is shown in Fig. 16.

**Table2:** Sensitivity analysis of operating time

Removed study	Heterogeneity test		Meta analysis	
	I^2^ (%)	P	MD	95% CI
Luo 2015	92	0.001	6.38	1.56–11.21
Zhang 2017	85	0.02	4.29	0.80–7.78
He 2018	91	0.01	7.03	1.48–12.57
Guo 2019	92	0.008	7.40	1.89–12.91
Liang 2020	92	0.007	6.82	1.83–11.80
Chen 2021	78	0.0002	8.06	3.80–12.32

## Discussion

This study showed that there were no statistically significant differences in age and BMI between the two groups, indicating that the two groups were comparable. There were no significant differences in blood loss, time of hospitalization, seroma, concomitant injury, mesh infection, recurrence, uterine prolapse, foreign body sensation, and chronic pain between the two groups. In terms of operation time, the preservation group took longer, and the difference was statistically significant. The above results fully indicate that due to the close adhesion of URL to the peritoneum, it does take more time to completely separate the URL from the peritoneum in the operation. However, there was no difference in the short-term and long-term complications after the operation. Therefore, whether the URL should be preserved during the operation was indeed a question worthy of in-depth discussion.

The total length of URL is 12–14 cm. It starts from the anterior horn of uterus, the anterior lower end of the proximal fallopian tube, under the cover of the anterior layer of the broad ligament of uterus, runs anterolateral, passes through the opening of the internal ring and the inguinal canal, and ends at the anterior end of the labia majora [22, 23]. It is composed of smooth muscle and connective tissue, and has no vascular distribution in it, which plays an important role in maintaining the forward position of the uterus [24].The URL is still an intraperitoneal organ at the corner of uterus, and gradually migrates into interperitoneal organs and extraperitoneal organs when it moves towards the opening of the internal ring [25]. The URL here is tightly attached to the transverse fascia of abdomen, which is difficult to be separated. Therefore, the problem of how to deal with URL is bound to be faced in the operation of groin repair for female patients.

In the early stage of laparoscopic inguinal hernia surgery, the preservation of URL increases the difficulty of the operation and cutting URL does not cause serious complications, inadequate protection of URL has been common in female groin hernia repair for a long time [6]. Due to the lack of understanding of the anatomy and function of URL, in the classic Shouldice procedure, the URL should be removed in order to close the inner ring.

As the function of URL is gradually being recognized, it is believed that its existence has its inevitable role. The reasons for preserving URL were as follows: (1) From the perspective of anatomy, the cut off URL will lose its role in maintaining the anteversion of uterus, and the retroposition of uterus can cause menstrual reflux, which is one of the causes of endometriosis [13]; (2) The cervix of the posterior uterus is raised, so that sperm can enter the uterine cavity is difficult, which can cause infertility [13]; (3) It can prevent uterine prolapse, the URL is not only important in women of childbearing age, but also plays an important role in elderly patients. Its retention can not only prevent pelvic organ prolapse, but also serve as a fulcrum for surgical treatment when diseases such as pelvic prolapse occur [26, 27]; (4) It has been reported that URL is not all tendinous structures, but contains muscular and tubular structures (lymphatic vessels) in the middle. Some scholars have confirmed that there was a far higher probability of edema of the labia majora after cutting URL. The reason may be that cutting URL will not only increase the local exudation, but also cause partial lymphatic reflux obstruction around the inner ring opening and the labia majora, resulting in increased flow in the surgical field and edema of the labia majora [1]. Based on the above reasons, the significance of preserving URL cannot be ignored, especially for women of childbearing age.

On the other hand, there are also more scholars advocate cutting URL, mainly for the following reasons: (1) IEHS (International Endoscopic Hernia Society) guidelines also suggest that in order for the patch to effectively cover the fascia defect, the URL should be cut, so that the patch can cover the pectineus foramina in a smooth way, so as to achieve complete repair of the pectineus foramina and reduce the recurrence of oblique inguinal hernia [4, 28]; (2) The peritoneum on the surface of URL is dense, so it is difficult to completely separate URL, which will cause postoperative bleeding and correspondingly prolong the operation time [13–15].

Whether cutting the URL increases the incidence of uterine prolapse was mentioned in only three studies in this meta-analysis. In Guo’s study [16], the follow-up period was 24–90 months. Uterine prolapse occurred in 5 patients in division group, but none in the preservation group. There was significant difference between the two groups. In Yan’s study[17], the mean follow-up time was 42.9 ± 37.3 months, and 1 case of uterine prolapse occurred in the division group and 3 cases in the preservation group, with no statistically significant difference between the two groups. In Liu’s study[19], the preservation group was followed up for 41.8 ± 24.2 months, and 4 cases of uterine prolapse occurred, while the division group was followed up for 42.7 ± 24.6 months, and 5 cases of uterine prolapse occurred, with no statistical significance between the two groups. In view of the fact that the occurrence of uterine prolapse requires a long follow-up time, the follow-up time of the above three studies is relatively short, and the conclusions are contradictory. Therefore, more studies with longer follow-up time should be included to draw more accurate conclusions.

Regarding the controversy about whether cutting the URL can cause infertility, few literatures have been reported, and only two studies have mentioned it [19, 20]. Due to the small amount of data, meta-analysis is not possible. Liu's study showed that after surgery, 14/218(6.42%) in the preservation group and 11/175(6.29%) in the division group had given birth or become pregnant, with an average follow-up of 41.8 months in the preservation group and 42.7 months in the division group [19]. The proportion of women in the two groups had similar natural childbirth. Although fertility and natural delivery rates are affected by many factors, they suggest that cutting URL has little effect on fertility [19]. Liang’s study showed that 13 patients (61.9%) in the preservation group and 6 patients (30.0%) in the division group had fertility needs, of which 6 patients (46.2%) in the preservation group and 4 patients (66.7%) in the division group were successfully pregnant and gave birth [20]. The results showed that there was no statistical significance in the fertility rate after surgery between the two groups [20]. However, it should be noted that some patients in the group with preservation URL had a short follow-up time after surgery and some patients did not intend to become pregnant, and this part of patients needed to be observed after extended follow-up. Liang believes that the preservation of URL is one less factor affecting infertility for women in the fertile period [20]. As far as the existing evidence is concerned, whether the preservation of URL has an impact on pregnancy or not, the results are not clear. We believe that for unmarried women who are not pregnant, the preservation of URL can be considered to avoid unnecessary trouble for patients.

The studies we reviewed were of relatively high quality. However, given some limitations, the results of our meta-analysis should be interpreted with caution. First, heterogeneity was found in meta-analyses of multiple variables (age, operating time, time of hospitalization, uterine prolapse and chronic pain), which may have reduced the reliability of these analyses, although we did compensate by using a random effects model. Second, some meta-analyses involve fewer patients, which may also affect their reliability. Third, only 3 randomized controlled studies were included. Fourth, all the included studies were Chinese studies, which may be biased. Fifth, despite an extensive literature search, we may have missed some unpublished studies.

In conclusion, laparoscopic groin hernia repair in women with the preservation of URL requires a longer operation time, and this systematic review and trial sequential analysis provide a conclusive evidence. Short-term or long-term complications were not significantly different between the two procedures, and there was no evidence that cutting URL caused infertility. More studies with longer follow-up were needed to draw more accurate conclusions about whether there was an increased incidence of uterine prolapse. Therefore, the author believes that for some unmarried women who are not pregnant, it can be considered to preserve URL, but these need to be further confirmed by larger prospective randomized controlled studies.
